# A Distinct Mechanism of Vascular Lumen Formation in *Xenopus* Requires EGFL7

**DOI:** 10.1371/journal.pone.0116086

**Published:** 2015-02-23

**Authors:** Marta S. Charpentier, Panna Tandon, Claire E. Trincot, Elitza K. Koutleva, Frank L. Conlon

**Affiliations:** 1 University of North Carolina McAllister Heart Institute, UNC-CH, Chapel Hill, North Carolina, United States of America; 2 Department of Genetics and Molecular Biology, UNC-CH, Chapel Hill, North Carolina, United States of America; 3 Department of Biology, UNC-CH, Chapel Hill, North Carolina, United States of America; 4 Lineberger Comprehensive Cancer Center, UNC-CH, Chapel Hill, North Carolina, United States of America; University of Oklahoma Health Science Center, UNITED STATES

## Abstract

During vertebrate blood vessel development, lumen formation is the critical process by which cords of endothelial cells transition into functional tubular vessels. Here, we use *Xenopus* embryos to explore the cellular and molecular mechanisms underlying lumen formation of the dorsal aorta and the posterior cardinal veins, the primary major vessels that arise via vasculogenesis within the first 48 hours of life. We demonstrate that endothelial cells are initially found in close association with one another through the formation of tight junctions expressing ZO-1. The emergence of vascular lumens is characterized by elongation of endothelial cell shape, reorganization of junctions away from the cord center to the periphery of the vessel, and onset of Claudin-5 expression within tight junctions. Furthermore, unlike most vertebrate vessels that exhibit specialized apical and basal domains, we show that early *Xenopus* vessels are not polarized. Moreover, we demonstrate that in embryos depleted of the extracellular matrix factor Epidermal Growth Factor-Like Domain 7 (EGFL7), an evolutionarily conserved factor associated with vertebrate vessel development, vascular lumens fail to form. While Claudin-5 localizes to endothelial tight junctions of EGFL7-depleted embryos in a timely manner, endothelial cells of the aorta and veins fail to undergo appropriate cell shape changes or clear junctions from the cell-cell contact. Taken together, we demonstrate for the first time the mechanisms by which lumens are generated within the major vessels in *Xenopus* and implicate EGFL7 in modulating cell shape and cell-cell junctions to drive proper lumen morphogenesis.

## Introduction

The formation of a functional vascular system during embryogenesis is critical for growth and survival. The development of a majority of organs and tissues first requires the proper establishment of a closed circulatory loop capable of transporting blood and nutrients, removing waste, and facilitating gas exchange. Blood vessels initially arise via vasculogenesis, which is characterized by the de novo assembly of endothelial precursors called angioblasts into primitive capillary-like networks [1,2,3]. Further expansion and remodeling of this primary plexus occurs by angiogenesis, or the sprouting of new vessels from preexisting vessels. Sprouting angiogenesis is the primary means by which a hierarchically branched and perfusable vascular system comprised of veins, arteries, and capillaries is ultimately formed [4,5,6,7,8].

Vascular development is well-conserved from fish to mammals. In addition to the presence of a pulmonary circulatory system, an inherent feature that makes *Xenopus* an ideal model in which to study the early events associated with endothelial cell assembly is their external development thus enabling one to easily visualize embryonic blood vessel formation with minimal physical manipulation. Some of the earliest vessels that arise via vasculogenesis in *Xenopus* include the paired posterior cardinal veins, the dorsal aorta, the vitelline vein network, the aortic arches, and the endocardium, all of which are derived from the mesoderm [9,10]. However, the cellular origins of these vessels differ as has been demonstrated by fate-mapping experiments, wherein by the 32-cell stage, the endothelial lineage becomes restricted to four blastomeres each of which give rise to different endothelial cell populations [11,12,13]. Angioblasts of the vitelline vein network form in close association with hematopoietic precursors in clusters known as the ventral blood islands whereas the dorsal lateral plate mesoderm (DLPM) contributes to the posterior cardinal veins and the dorsal aorta [13].

The posterior cardinal veins and the dorsal aorta constitute the major embryonic vessels and begin forming shortly after neurulation [14]. DLPM angioblasts first align into two bilateral strips in an anterior-to-posterior direction beginning at late neurula stage (stage 20) [11]. During tailbud stages (stage 27–32), a subset of these cells migrates over the underlying endoderm to the midline and settles directly beneath the hypochord to ultimately form the dorsal aorta [14,15]. It has been shown that this wave of angioblast migration occurs in response to vascular endothelial growth factor (VEGF) expressed by the hypochord [14]. It is still unclear whether DLPM cells are the sole contributors to the dorsal aorta in *Xenopus* as studies in chick have also demonstrated a contribution from somitic mesoderm [16,17]. Migration is completed by late tailbud stage (stage 33/34) thereby specifying the positions where the posterior cardinal veins and dorsal aorta will differentiate. Tubular vessels become apparent beginning at early tadpole stage (stage 35/36) concomitant with the commencement of circulation.

Very few studies to date have examined how vascular lumens are generated in *Xenopus*, although Vokes and colleagues demonstrated that tube formation, but not angioblast specification, requires the endoderm [18]. Indeed, vascular lumen formation is still an incompletely understood process in other organisms as well. Studies using cultured human endothelial cells, zebrafish, and mouse have elucidated some important molecular determinants of vascular lumen formation [19,20,21,22,23,24,25,26]. The predominant mechanism by which lumens are generated has been termed cord hollowing or extracellular lumen formation referring to a cell-cell separation event that results in a luminal compartment contained between multiple cells [27,28,29]. Previous studies in the mouse elegantly demonstrated that endothelial cells of the dorsal aorta initially adhere to each other through the formation of multiple junctions along the contact surface [22]. Prior to cell-cell separation, these vascular cords establish apicobasal polarity and it is hypothesized that this polarization event drives the redistribution of junctions away from the cord center to the periphery of the vessel [22,30]. Furthermore, Rho kinase (ROCK)-dependent signaling facilitates the appropriate cell shape changes needed to accommodate the developing lumen. Similar cord hollowing mechanisms have been observed in zebrafish vessels; however, additional mechanisms including intracellular lumen formation via coalescence of pinocytic vacuoles or apical membrane invagination as well as lumen ensheathment have also been demonstrated thus signifying that different species and/or different vascular beds may use diverse ways to generate and maintain vascular lumens [21,23,24,25,28,31,32].

Epidermal Growth Factor-Like Domain 7 (EGFL7) is a small protein of 20–30 kD that is highly expressed in endothelial cells actively engaged in vessel assembly during embryonic development, injury, pregnancy, and tumorigenesis [33,34,35,36,37,38,39]. Structural analysis of EGFL7 revealed it to contain an Emilin-like domain characteristic of secreted proteins and indeed, EGFL7 is secreted by endothelial cells and deposited into the extracellular matrix (ECM) [33,40]. Numerous studies have implicated EGFL7 in vascular lumen morphogenesis as well as sprouting angiogenesis. In zebrafish, EGFL7 was shown to be required for the establishment of vascular lumens likely by providing a permissive environment for endothelial cell adhesion and migration [35]. In accordance with these studies, we have shown that EGFL7 is also required for vascular lumen formation in *Xenopus* and modulates endothelial cell behaviors including cell shape and adhesion to promote vessel sprouting [41]. Overexpression studies in mouse have further indicated that EGFL7 plays a role in vessel patterning and remodeling potentially through its interaction with Notch receptors [42]. Finally, EGFL7 is currently being investigated in Phase II clinical trials by Genentech for its role in promoting tumor angiogenesis. The company has developed monoclonal antibodies against EGFL7 which have been shown to augment the efficacy of anti-VEGF therapies in pruning and damaging tumor vessels in a model of non-small cell lung cancer [43]. Thus, understanding the function and regulation of this protein is of therapeutic value.

For the first time, we demonstrate the cellular and molecular mechanisms underlying vascular lumen formation in *Xenopus* using the posterior cardinal veins and the dorsal aorta as models. While lumens do appear to be generated via cord hollowing, we note some distinct features in *Xenopus* such as the absence of apicobasal polarity. Furthermore, we investigated the underlying defects associated with impaired lumen formation in EGFL7-depleted embryos and determined that cells fail to undergo proper cell shape changes and reorganize cell-cell junctions in order to accommodate the luminal compartment.

## Materials and Methods

### *Xenopus* embryo manipulation and collection

*Xenopus* embryos were prepared and collected as previously described [44]. Embryos were staged according to Nieuwkoop and Faber [45]. *Egfl7* and control morpholinos were used as previously described [41,46]. Five hundred pg of mRNA encoding membrane-GFP/pCS2 (Construct kindly provided by John Wallingford lab, UT-Austin) were co-injected with 35 ng of *Egfl7* or control morpholino into 1-cell stage embryos. For explant assays, the anterior region of stage 28 embryos consisting of the head and heart was removed using sterilized forceps. Explants were cultured for 24 hours in 0.1X Modified Barth's Solution (MBS) containing 10 μg/mL gentamycin before fixation. xFlk-1:GFP transgenic frogs were a kind gift from P.E. Mead [47].

### Whole mount in situ hybridization

In situ hybridization was carried out as previously described using an anti-sense probe against *Ets-related gene* (*Erg)* [41,48]. For experiments in which in situ hybridization was coupled with immunohistochemistry, changes to the published in situ protocol were made as follows: 1) Embryos were fixed in 4% paraformaldehyde (PFA) for two hours at room temperature or 4° overnight then stored in 1X phosphate buffered solution (PBS); 2) Fixed embryos were rinsed three instead of five times in PBS-T (0.1% Tween-20) prior to Proteinase K treatment; 3) Embryos were incubated in 10 μg/mL Proteinase K (Roche) for 10 minutes; 4) Embryos were rinsed once in PBS-T following Proteinase K treatment and immediately fixed in 4% PFA for 20 minutes; 5) Following color reaction in BM Purple (Roche), embryos were washed three times in 1X PBS and immediately processed for histology and immunostaining.

### Histology

For time course analysis of lumen formation, in situ hybridization was performed as previously described without the above modifications [48]. After completion of BM purple color reaction, embryos were taken through a methanol:PBS gradient (five minutes each in 25% methanol:75% PBS, 50% methanol:50% PBS, 75% methanol:25% PBS) and dehydrated in 100% methanol for at least 24 hours. Embryos were subsequently rehydrated in PBS and taken through a PBS:glycerol gradient (24 hours each in 25% glycerol:75% PBS, 50% glycerol:50% PBS, 75% glycerol:25% PBS, 100% glycerol). Embryos were incubated in a gelatin solution (0.4% Gelatin Type A [Sigma], 27% Bovine Serum Albumin [Sigma], 18% sucrose, PBS) for 24 hours and then embedded in the gelatin mixed with 25% glutaraldehyde (Grade II, Sigma). A Leica VT1200S vibratome was used to cut 20 μm transverse sections. Sections were directly mounted onto slides and imaged on an Olympus IX81 inverted fluorescent microscope. For immunohistochemistry, following the modified in situ protocol the head and the posterior trunk including the tail were first removed and the anterior trunk region was used for sectioning to ensure more consistent and comparable analysis across sections as formation of the vessels occurs in an anterior-to-posterior direction [9,14]. The anterior trunk regions were embedded in 4% low melting point agarose (Promega) made in 1X PBS and a Leica VT1200S vibratome was used to cut 70 μm transverse sections that were collected in 0.5X PBS. For xFlk-1:GFP studies, 100 μm thick transverse vibratome sections were collected.

### Immunohistochemistry and imaging

Immunohistochemistry (IHC) was carried out on sections post-in situ hybridization in a 48-well plate beginning with three 30-minute PBS-T (1% Triton X-100) washes followed by incubation for one hour in PBS-T containing 10% fetal bovine serum (FBS). Primary antibodies as listed in S1 Table were applied overnight at 4° (Laminin IHC was performed on sections not previously processed by in situ hybridization). The following day, sections were washed five times for one hour each in PBS-T before incubation with secondary antibodies as listed in S1 Table overnight at 4°, rinsed five times in PBS-T, incubated with 200 ng/mL DAPI/PBS solution (Sigma) for 30 minutes, and mounted. For F-actin staining, sections that had not been processed by in situ hybridization were washed and blocked as above and incubated with Phalloidin-Alexa Fluor 488 (Molecular Probes A12379, 1:100 in DAPI/PBS solution) for 30 minutes. Images and Z-stacks were taken with a Zeiss 700 confocal microscope using a 63X oil objective. For Z-stacks, slices were imaged at 1 μm intervals and stacks were reconstructed to represent 1–3 μm projected images using ImageJ. The luminal area of the posterior cardinal veins was measured using ImageJ.

### Ethics statement

This study was carried out in strict accordance with the recommendations in the Guide for the Care and Use of Laboratory Animals of the National Institutes of Health. The protocol (#13–260) was approved by the IACUC committee of the University of North Carolina at Chapel Hill. *Xenopus laevis* embryos were used to perform in vivo assays described above. Embryos were obtained by *in vitro* fertilization of *Xenopus laevis* eggs laid by adult females primed with 1000U Chorulon (human chorionic gonadotrophin hormone). Females were allowed to recover for 2–3 months before priming again. Testes were collected from euthanized (2 g/L tricaine methane sulfonate [Argent Labs] + 2g/L sodium bicarbonate in water) adult males, as needed. A single female will lay thousands of eggs on a given day after being primed. Enough eggs were fertilized to provide sufficient numbers of embryos for each type of assay. Care was taken to ensure that animals did not experience any discomfort during any of the procedures outlined above. All euthanasia was carried out according to IACUC protocols. All procedures are consistent with the recommendations of the Panel of Euthanasia of American Veterinary Medical Association.

## Results

### Lumens of the major veins and artery arise simultaneously in *Xenopus*

Lumen formation has been reported to occur through a variety of mechanisms in different vertebrate model systems. *Xenopus* offers a unique perspective in this regard since it is evolutionarily positioned between animals that have an undivided circulation, i.e. zebrafish and those vertebrates that have a separate pulmonary and systemic circulation. To investigate the mechanisms underlying vascular lumen formation in *Xenopus*, we conducted a time course during vessel development to determine when vascular lumens arise. Our studies focused on three major vessels, the dorsal aorta as an example of arterial endothelial cell types, and the paired posterior cardinal veins as examples of the venous endothelial cell types.

**Fig 1:**
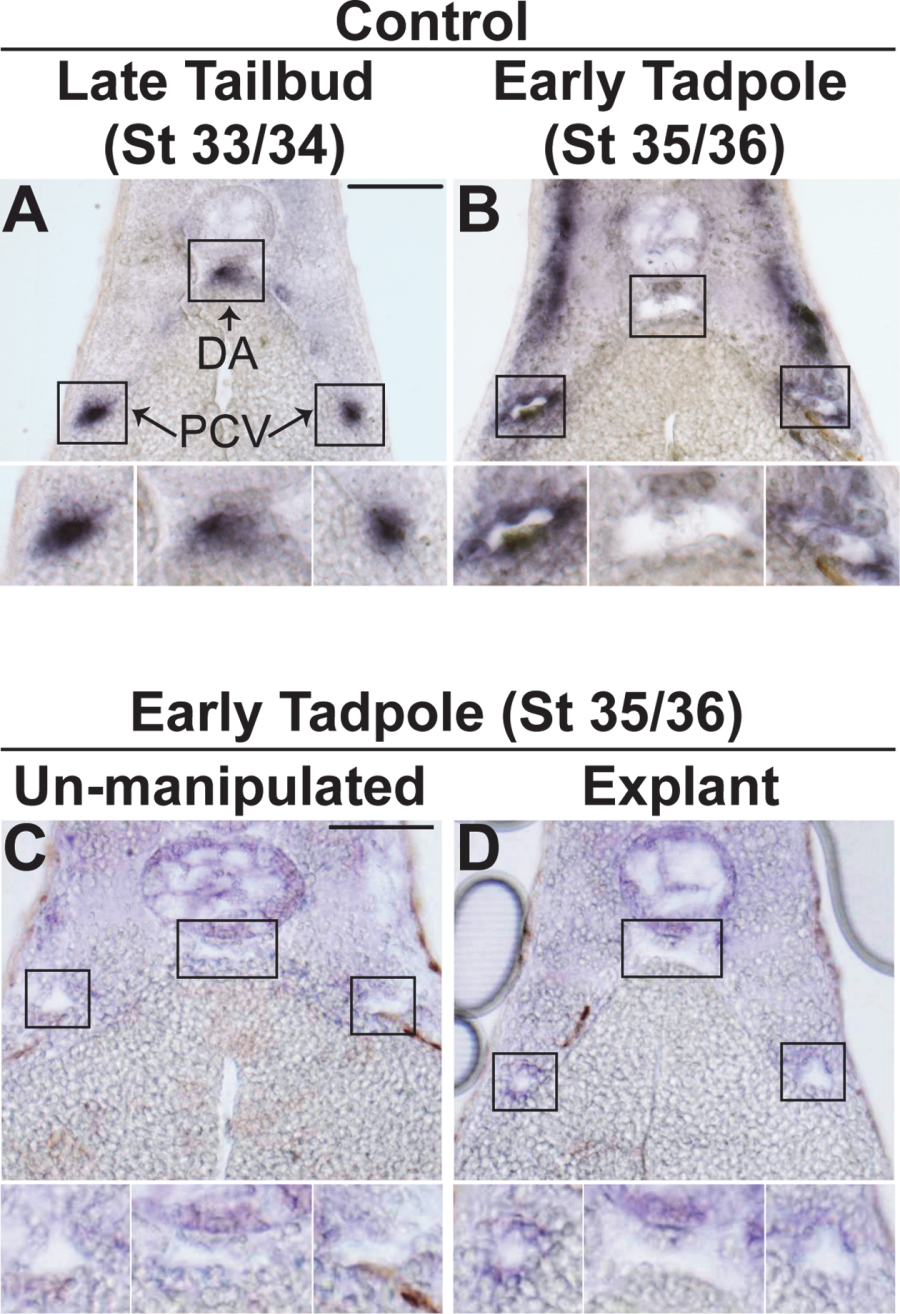
Vascular lumen formation proceeds independently of circulation in *Xenopus*. (**A-B**). In situ hybridization with endothelial-specific *Erg* followed by sectioning was performed to determine the time course of vascular lumen formation of the dorsal aorta (DA) and posterior cardinal veins (PCV) in *Xenopus*. (**A**). Endothelial cells are coalesced together at the correct positions of the DA and PCVs in late tailbud stage (stage 33/34) control embryos. (**B**). Lumenized DA and PCVs become apparent by early tadpole stage (stage 35/36) in control embryos. 3–4 embryos from each condition/stage were assessed from at least three independent injection batches at the same position along the anterior-posterior axis of the embryo. (**C-D**). In situ hybridization with *Erg* followed by sectioning. (**C**). Transverse section of an early tadpole stage (stage 35/36) wildtype embryo depicting lumenized DA and PCVs. (**D**). Following the removal of the anterior and heart of early tailbud embryos, explants were cultured until early tadpole stage (stage 35/36) and processed as indicated above. PCV and DA lumens readily form in the absence of a functioning heart or circulatory system. 3–4 embryos from each condition were assessed from two independent experiments. Top image of each panel was taken at 20x magnification. Black boxes correspond to enlarged images of each vessel displayed below. Scale bars represent 20 μm.

In *Xenopus* just prior to mid-tailbud stage, endothelial cells of the dorsal lateral plate mesoderm coalesce in two bilateral strips corresponding to the positions of the posterior cardinal veins [11,12]. Shortly thereafter, a subset of these angioblasts migrates over the underlying endoderm towards the midline. We found that this group of cells, as marked by endothelial-specific *Ets-related gene* (*Erg*), begin to coalesce just dorsal to the hypochord to form the dorsal aorta (Fig. 1A). The wave of migration is completed by late tailbud stage (stage 33/34) when all three populations corresponding to the endothelial cells of the posterior cardinal veins and the dorsal aorta can be distinguished. However, at this stage no lumens can be detected (Fig. 1A). By early tadpole stage (stage 35/36), a time corresponding to E7.5-E8.0 in mouse and 18±1 days in human, vascular lumens of both the veins and the aorta become evident (Fig. 1B). These data imply that lumenization of the posterior cardinal veins and the aorta occurs simultaneously by early tadpole stages.

### Circulation is not essential for lumen formation

Lumen formation is often associated with the onset of a functional circulatory loop. Furthermore, it has been postulated that circulation is required for vessel remodeling and stabilization [49,50,51]. Therefore one hypothesis is that lumen formation is a function of vascular flow. To address this possibility, we took advantage of the ability to culture *Xenopus* embryo explants for extended periods of time in culture [41,52,53], removing the anterior-most region of embryos inclusive of the heart at a stage prior to cardiac development (i.e. stage 28). From these studies we found that the loss of the cardiac region of the embryo has no effect on the migration, aggregation or critically, the lumenization of either the posterior cardinal veins or aorta (Fig. 1C-D). Thus, these studies imply that cardiac development and the establishment of circulation are not prerequisites for the initial establishment of vascular lumens in *Xenopus*.

### Early *Xenopus* vessels are not characterized by distinct apical and basal domains

**Fig 2:**
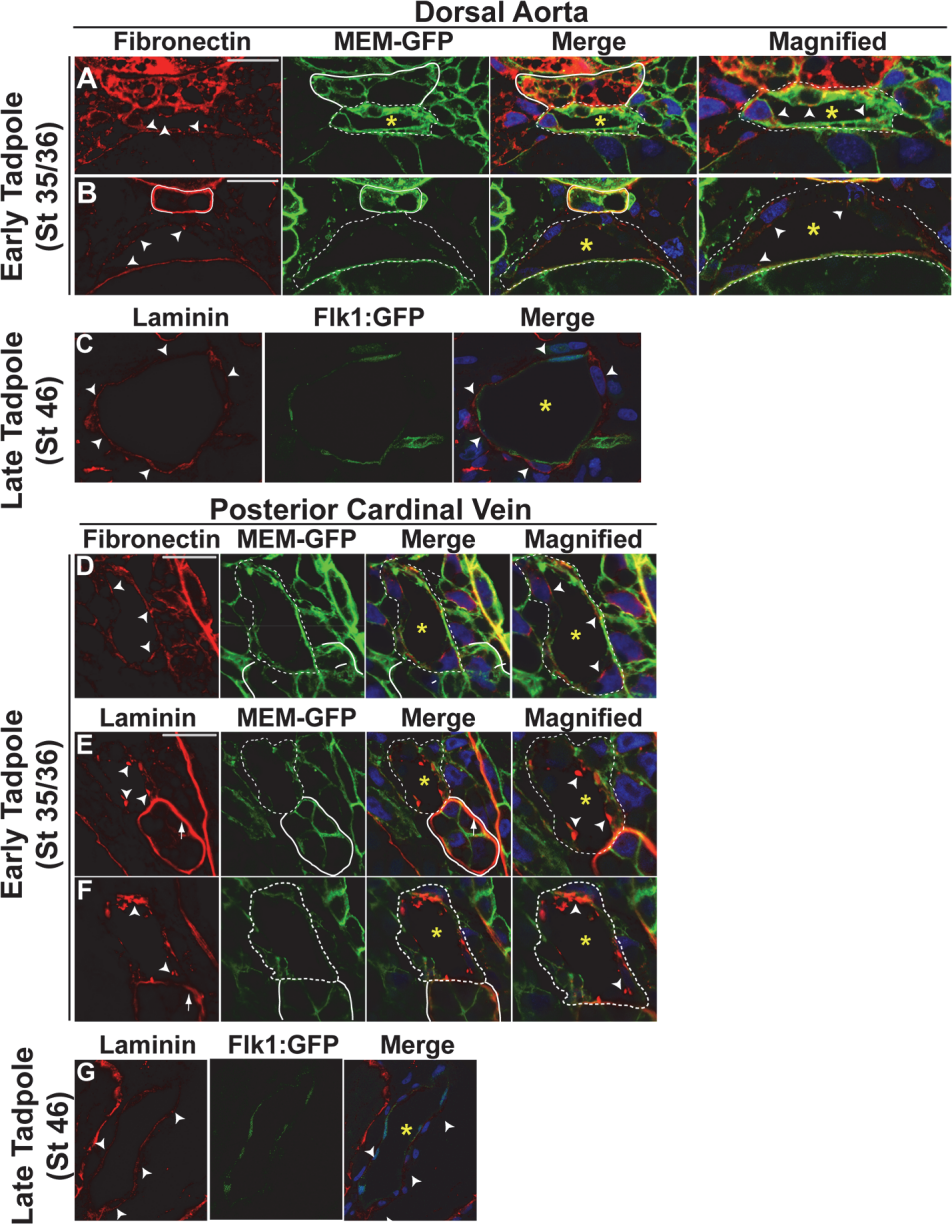
Basement membrane proteins are deposited on both the basal and apical surfaces of early vessels in *Xenopus*. **(A-G)** Representative confocal images of transverse sections of the dorsal aorta (DA) and posterior cardinal veins (PCV) stained with laminin or fibronectin (red). In A,B,D,E,F staining with GFP to mark cell membranes (MEM-GFP) and DAPI to mark nuclei (blue) is also shown. In C and G, Flk-1:GFP transgenic animals were used to identify endothelial cells (green). All images taken at 63x magnification with scale bars indicating 20 μm. Dotted white lines in all panels delineate the endothelial cells comprising each vessel. As anatomical references, the hypochord in (A-B) and the kidney in (D-F) are indicated by solid white lines. Dotted lines in the magnified images represent vessels. Asterisks denote the vessel lumen. (**A**). The DA of early tadpole stage (stage 35/36) control embryos display deposition of fibronectin on both apical and basal surfaces (arrowheads point to apical staining). (**B**). DA of early tadpole stage (stage 35/36) control embryo exhibits the laminin staining on the basal and the apical surfaces of the vessel (arrowheads point to apical staining). (**C**). By late tadpole stage (stage 46), proper polarity is established in the dorsal aorta with laminin localizing to the basal surface of endothelial cells (arrowheads point to basal staining). (**D**) PCV of early tadpole stage (stage 35/36) control embryo exhibits apically and basally deposited fibronectin (arrowheads point to apical staining). (**E-F**). The kidney displays appropriate basal expression of laminin (arrow), however, the PCV of early tadpole stage (stage 35/36) control embryos display aggregates of laminin staining on the apical surface (arrowheads). (**G**) Laminin becomes distributed on the basal surface of the posterior cardinal vein in late tadpole stage (stage 46) embryos (arrowheads point to basal staining). 3–4 embryos from each condition/stage were assessed from at least three independent injection batches at the same position along the anterior-posterior axis of the embryo.

The establishment of apicobasal polarity has been found to be associated with lumen formation in zebrafish and mouse [22,27,54,55]. To test the role of polarity in *Xenopus* lumen formation, we examined the distribution of proteins known to be enriched on either the basal or apical surface of blood vessels in zebrafish and mouse. We first examined fibronectin, an ECM marker with preferential basal localization in vessels [56,57,58,59], and found little enrichment on the basal surface of endothelial cells of the aorta and veins of stage 35/36 embryos (Fig. 2A,D). In some cases fibronectin appeared along the apical surface of endothelial cells, particularly in the veins (Fig. 2D) but overall was not extensively polarized. Consistent with these results laminin, a marker of basement membrane [60,61,62], also failed to preferentially localize to the basal surface of the aorta and veins in stage 35/36 embryos although basal localization was evident in the kidney (Fig. 2B,E,F). However, deposits of laminin were observed on the apical surface of venous endothelial cells implying that early *Xenopus* vessels lack apicobasal character despite the presence of a lumen. We next examined later tadpole stages (stage 46) when remodeling of the embryonic vasculature is complete and the paired posterior cardinal veins have fused into a single vessel [10,47]. Interestingly, we found that laminin was exclusively localized to the basal surface of endothelial cells of both the aorta and the vein (Fig. 2C,G). Taken together, these results imply that formation of a primary vascular lumen does not necessarily coincide with the establishment of basal polarity, a characteristic that is clearly indicative of further remodeled, mature vessels.

**Fig 3:**
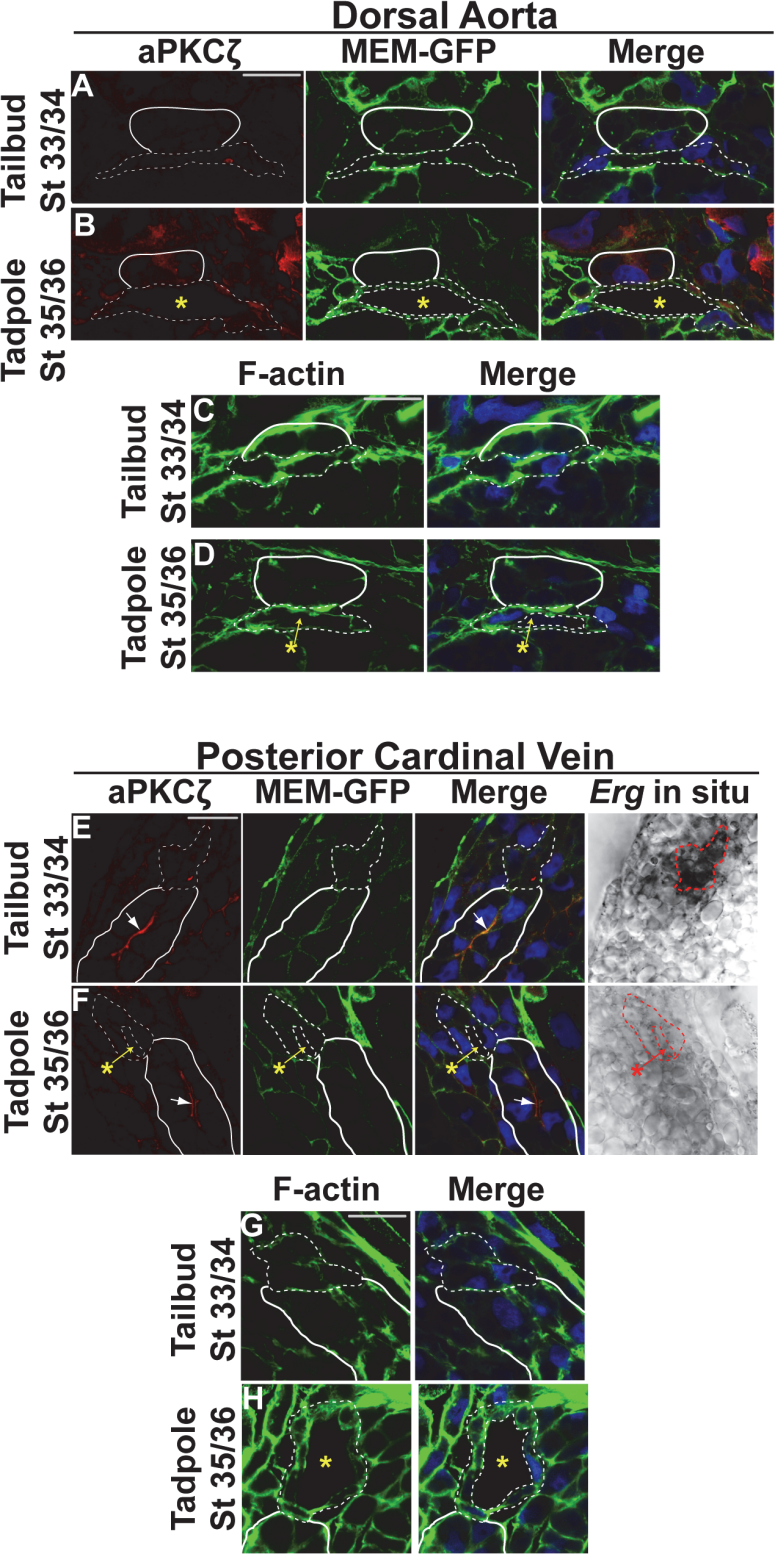
Apical polarity is not established in *Xenopus* vessels. (**A-H**). Representative confocal images of transverse sections of the dorsal aorta (DA) and posterior cardinal veins (PCV) stained with atypical PKCζ (aPKCζ; red), GFP to mark cell membranes (MEM-GFP; green), and DAPI to mark nuclei (blue; A,B,E,F) or Phalloidin to mark F-actin (green) and DAPI (blue; C,D,G,H). Phase contrast images for the PCVs in (E) and (F) represent the *Erg* in situ signal. All images taken at 63x magnification with scale bars indicating 20 μm. Dotted white and red lines in all panels delineate the endothelial cells comprising each vessel. As anatomical references, the hypochord in (A-D) and the kidney in (E-H) are indicated by solid white lines. Asterisks denote the vessel lumen. (**A-B**). aPKCζ enrichment is absent from the cell-cell contact in late tailbud stage (stage 33/34) control embryos and the apical membrane of the DA of early tadpole stage (stage 35/36) control embryos. 3–4 embryos from each condition/stage were assessed from at least three independent injection batches at the same position along the anterior-posterior axis of the embryo. (**C-D**). F-actin fails to localize to the cell-cell contact or apical surface of the dorsal aorta at late tailbud and early tadpole stages. (**E-F**). aPKCζ can readily be detected on the apical surface of the kidney (arrow) but not at the cell-cell contact or apical membrane of the PCV at late tailbud (stage 33/34) or early tadpole stages (stage 35/36). (**G-H**). F-actin is evenly distributed in venous endothelial cells. 3–4 embryos from each condition/stage were assessed from at least two independent injection batches at the same position along the anterior-posterior axis of the embryo.

To confirm and extend these findings, we examined the expression of the apical marker, atypical protein kinase C zeta (aPKCζ) [54,63,64,65]. Although localization of apical proteins along the cell-cell contact even prior to lumen formation has been shown to be apparent in mouse [22], we found that in *Xenopus* aPKCζ fails to localize to the apical surface of endothelial cells of the dorsal aorta before lumen formation (stage 33/34; Fig. 3A). We also failed to detect aPKCζon the apical membrane of the lumenized dorsal aorta at stage 35/36 (Fig. 3B). While aPKCζ is localized to the apical surface of the kidney, we could not detect enrichment of aPKCζ on the apical or basal surface of endothelial cells of the veins prior to (stage 33/34) or after (stage 35/36) lumen formation (Fig. 3E,F). F-actin has also been demonstrated to localize to the cell-cell contact and the apical surface before and following dorsal aorta lumen formation in mouse due to its linkage to apical proteins including CD34-sialomucins such as PODXL and moesin [22,66,67]. Consistent with our observations for aPKCζ, we further observed that F-actin enrichment to the cell-cell contact in stage 33/34 control embryos is not evident (Fig. 3C,G). F-actin also fails to localize to the apical surface of the aorta or veins by stage 35/36 (Fig. 3D,H). Since we could not detect the organization of laminin, fibronectin, aPKCζ, or F-actin to distinct basal or apical domains, these findings would imply that early *Xenopus* vessels lack apicobasal character.

### EGFL7 is required for vascular lumen formation

**Fig 4:**
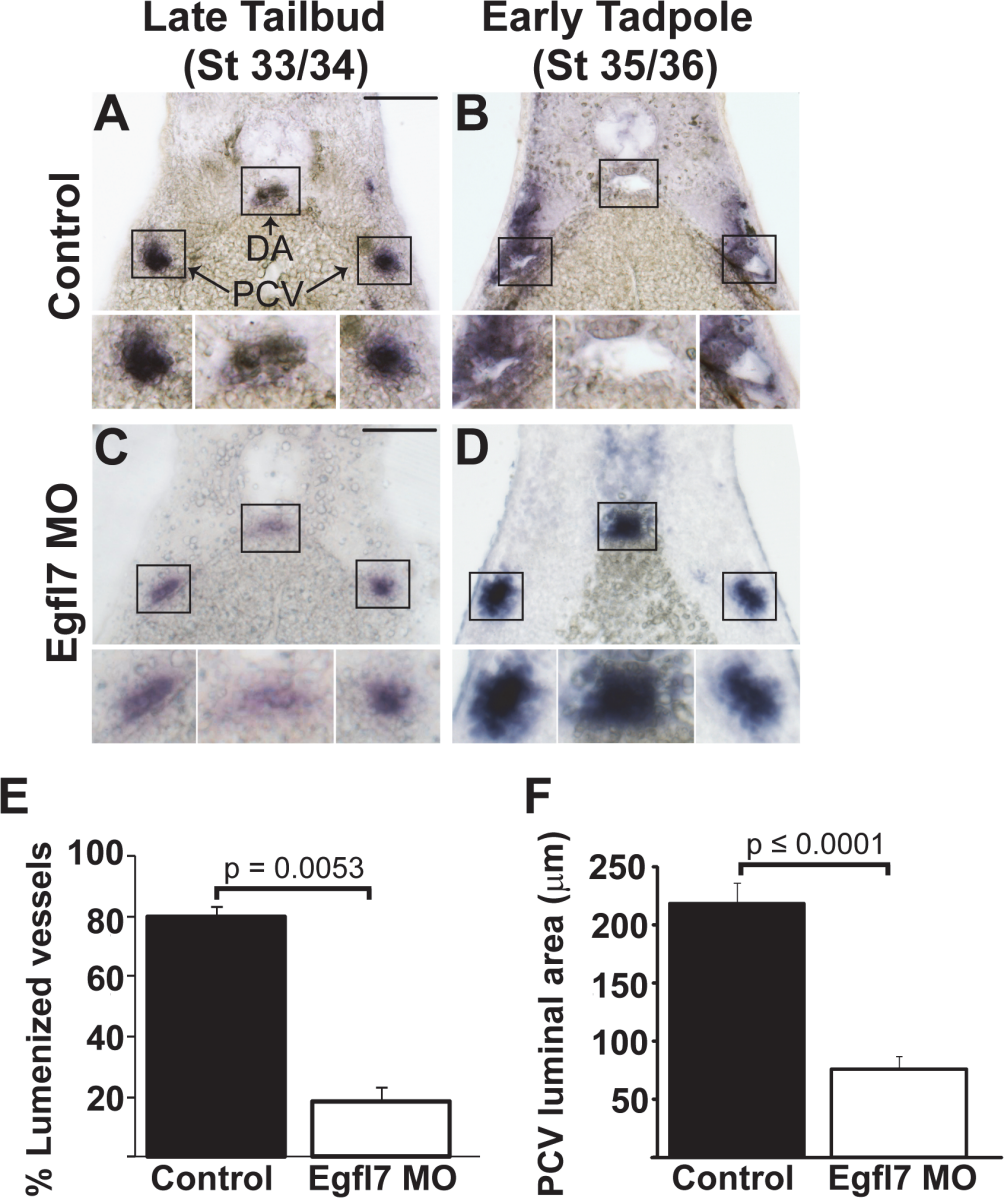
EGFL7 is required for lumen formation. (**A-D**). In situ hybridization with endothelial-specific *Erg* shows a requirement for EGFL7 in lumen formation of the dorsal aorta (DA) and posterior cardinal veins (PCV). (**A**). Endothelial cells coalesce at the appropriate positions of the DA and PCVs in late tailbud stage (stage 33/34) control embryos. (**B**). DA and PCV lumens become evident by early tadpole stage (stage 35/36) in control embryos. (**C**). Endothelial cells appear as aggregates in late tailbud stage (stage 33/34) EGFL7-depleted vessels similar to controls. (**D**). Lumens fail to form in early tadpole stage (stage 35/36) EGFL7-depleted embryos. 3–4 embryos from each condition/stage were assessed from at least three independent injection batches at the same position along the anterior-posterior axis of the embryo. Top image of each panel was taken at 20x magnification. Black boxes correspond to enlarged images of each vessel displayed below. Scale bars represent 20 μm. (**E**). Frequency of lumenized PCVs and DAs in early tadpole stage (stage 35/36) embryos. Lumens are detected in 81% of control embryos vs. 21% of EGFL7-depleted embryos. n = 209 control, n = 216 MO, three independent experiments. Student's t-test was used to calculate the p-value and bars represent ± SEM. (**F**). Measurement of the luminal area within the PCV in early tadpole stage (stage 35/36) embryos. The size of control lumens = 218 μm. Of EGFL7-depleted vessels that were lumenized, lumens were significantly smaller,75 μm. n = 70 control, n = 29 MO. A Mann-Whitney test was used to determine significance and bars represent ± SEM.

The findings that *Xenopus* lumen formation is not associated with flow or apicobasal polarity led us to investigate the function of the ECM-associated protein Epidermal Growth Factor-Like Domain 7 (EGFL7) in *Xenopus*. EGFL7 has been reported to be expressed and required for lumen formation in zebrafish and *Xenopus* [35,41,68]. However, the mechanisms by which EGFL7 acts in lumen formation are yet to be established. Therefore, we sought to determine the precise cellular function of EGFL7 in *Xenopus* lumen formation in embryos lacking EGFL7. From these studies, we found that migration and localization of endothelial cells to the positions of the veins and the aorta are indistinguishable between control and EGFL7-depleted embryos at stage 33/34 (Fig. 4A,C), thus suggesting that EGFL7 is not required for specification, migration or the coalescence of endothelial cells to the locations of the veins and aorta. However, at slightly later stages (stage 35/36) we observe significant differences in vascular development between controls and EGFL7-depleted embryos. Contrary to the well-developed vascular lumens of control vessels (Fig. 4B), endothelial cells of both the aorta and veins in EGFL7-depleted embryos remain clustered and fail to generate discernible lumens (Fig. 4D). These data suggest that EGFL7 acts at the initial stages of lumen morphogenesis in both the arterial and venous system.

To investigate the cellular dynamics of lumen formation in *Xenopus*, we introduced GFP tagged to CAAX into one-cell stage embryos to allow the visualization of cell membranes (MEM-GFP) [69]. As the presence or absence of a lumen can be easily deciphered based on the MEM-GFP staining, we quantified the percentage of control and EGFL7-depleted embryos that displayed lumenized vessels by stage 35/36. While both vein and aorta lumens were detected in 81.4% of control embryos by stage 35/36, only 21.3% of EGFL7-depleted embryos had undergone lumen formation by this time (Fig. 4E). In addition, of EGFL7-depleted embryos that did exhibit some sort of venous luminal compartment, the area of the lumen was significantly smaller compared to control embryos (Fig. 4F; 218 μm in control vs. 75.4 μm in EGFL7-depleted).

### EGFL7 is required for endothelial cell elongation during lumen formation

Lumen formation in the dorsal aorta in mouse has previously been shown to proceed via dynamic coordinated changes in endothelial cell behaviors including alterations in cellular morphology [22]. Prior to the emergence of a vascular lumen, endothelial cells coalesce into a cord-like structure. As the lumen is generated, the endothelial cells undergo elongation and take on a narrow shape to accommodate the developing luminal compartment [22]. Given our findings that embryos lacking EGFL7 fail to initiate lumen formation, we next investigated the role of EGFL7 in regulating the morphogenetic movements associated with the first steps in lumen formation.

**Fig 5:**
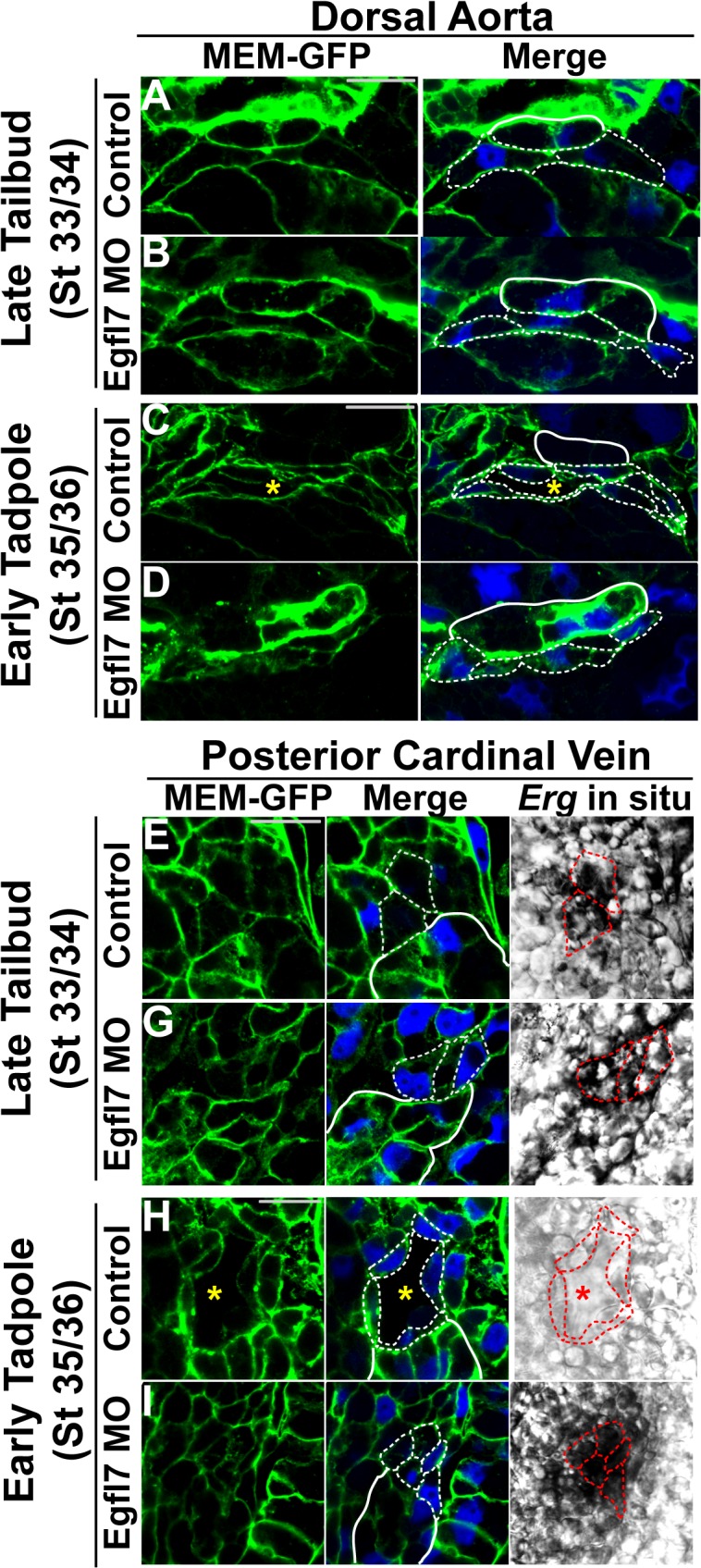
Cellular morphology of endothelial cells undergoing lumen formation. (**A-H**). Representative confocal images of transverse sections of the dorsal aorta (DA) and posterior cardinal veins (PCV) stained with GFP to mark cell membranes (MEM-GFP; green) and DAPI to mark nuclei (blue). Phase contrast images for the PCVs represent the *Erg* in situ signal. All images taken at 63x magnification with scale bars indicating 20 μm. Dotted white and red lines in all panels delineate individual endothelial cells. As anatomical references, the hypochord in (A-D) and the kidney in (E-H) are indicated by solid white lines. (**A**). Endothelial cells of the DA are oblong-shaped at late tailbud stage (stage 33/34) control embryos. (**B**). Endothelial cells of late tailbud stage (stage 33/34) EGFL7-depleted DAs display a similar morphology to controls. (**C**). Endothelial cells elongate during DA lumen formation in early tadpole stage (stage 35/36) control embryos. (**D**). Endothelial cells of the DA remain oblong-shaped and do not elongate to accommodate the vascular lumen in early tadpole stage (stage 35/36) EGFL7-depleted embryos. (**E**). Endothelial cells of the PCVs have a polygonal cobblestone-like shape in late tailbud stage (stage 33/34) control embryos. (**F**). Endothelial cells of late tailbud stage (stage 33/34) EGFL7-depleted PCVs are similarly shaped as controls. (**G**). Endothelial cells elongate, becoming narrower as PCV lumens form by early tadpole stage (stage 35/36) control embryos. (**H**). Endothelial cells of the PCVs retain their polygonal morphology in early tadpole stage (stage 35/36) EGFL7-depleted embryos. 3–4 embryos from each condition/stage were assessed from at least three independent injection batches at the same position along the anterior-posterior axis of the embryo.

Using MEM-GFP as a marker of cell membranes, we observed that the endothelial cells of the aorta are juxtaposed to the hypochord and display an oblong shape in control and EGFL7-depleted embryos at stage 33/34 (Fig. 5A,B). By stage 35/36, we found that endothelial cells of control embryos change shape appearing as thin, elongated cells (Fig. 5C). In stark contrast, arterial endothelial cells of EGFL7-depleted embryos appear to arrest morphogenetic movements at stage 33/34 and fail to undergo any further changes in cell shape (Fig. 5D). These data suggest that EGFL7 is required for modulating the morphological events associated with lumen formation.

To determine if the role of EGFL7 in mediating cellular elongation associated with lumen formation is unique to arterial endothelial cells, we further examined the role of EGFL7 in the posterior cardinal veins. At the stage prior to lumen formation (stage 33/34), control and EGFL7-depleted venous endothelial cells are located adjacent to the developing kidney and display a polygonal cobblestone-like morphology (Fig. 5E,F). Similar to the aorta, the venous endothelial cells at stage 35/36 elongate and become narrower to accommodate the formation of the lumen (Fig. 5G). Conversely, the morphology of EGFL7-depleted venous endothelial cells at stage 35/36 fail to undergo cell shape changes (Fig. 5H). Of note, examination of fibronectin revealed no discernible differences in ECM deposition between EGFL7-depleted embryos and controls suggesting that processes other than matrix remodeling are dependent on EGFL7 function (S1 Fig.). Taken together these results argue that EGFL7 is required within both venous and arterial endothelial cells to regulate the cell shape changes associated with lumen formation.

### EGFL7 is required for clearing junctions away from the cord center

**Fig 6:**
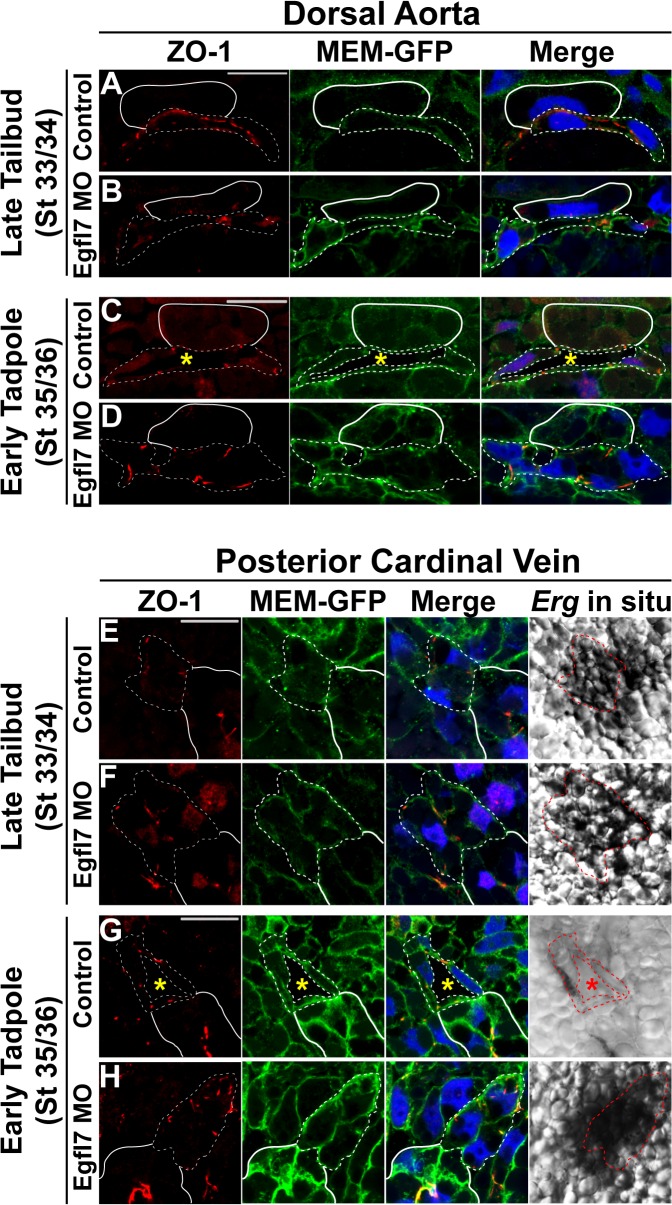
EGFL7 is required for proper reorganization of tight junctions. (**A-H**). Representative confocal images of transverse sections of the dorsal aorta (DA) and posterior cardinal veins (PCV) stained with ZO-1 (red), GFP to mark cell membranes (MEM-GFP; green), and DAPI to mark nuclei (blue). Phase contrast images for the PCVs represent the *Erg* in situ signal. All images taken at 63x magnification with scale bars indicating 20 μm. Dotted white and red lines in all panels delineate the endothelial cells comprising each vessel. As anatomical references, the hypochord in (A-D) and the kidney in (E-H) are indicated by solid white lines. (**A**). ZO-1 localizes to tight junctions between endothelial cells of the DA in late tailbud stage (stage 33/34) control embryos. (**B**). ZO-1 tight junctions between endothelial cells of the DA in late tailbud stage (stage 33/34) EGFL7-depleted embryos appear similar to controls. (**C**). Tight junctions are redistributed to distinct puncta between endothelial cells at the periphery of the lumen in early tadpole stage (stage 35/36) control embryos. (**D**). Tight junctions are retained between endothelial cells of the DA in early tadpole stage (stage 35/36) EGFL7-depleted embryos. (**E**). Tight junctions assemble along the cord center between endothelial cells of the PCV in late tailbud stage (stage 33/34) control embryos. (**F**). Similar to controls, tight junctions assemble between adjacent endothelial cells of the PCV in late tailbud stage (stage 33/34) EGFL7-depleted embryos. (**G**). Tight junctions are redistributed to the periphery of the lumenized PCV and appear as distinct points of cell-cell contact in early tadpole stage (stage 35/36) control embryos. (**H**). Tight junctions are retained along the cord center of the PCV in early tadpole stage (stage 35/36) EGFL7-depleted embryos. 3–4 embryos from each condition/stage were assessed from at least 3–4 independent injection batches at the same position along the anterior-posterior axis of the embryo.

Reorganization of cell junctions has been shown to be a critical event underlying vascular lumen formation [22,23,24,32]. We therefore examined the distribution of junctions prior to and following lumen formation in *Xenopus* in the context of EGFL7-depletion. Zonula occludens-1 (ZO-1) is a well-established marker of tight junctions shown to localize to cell-cell contacts during the early stages of vessel assembly in zebrafish [24,32,56,70]. In the dorsal aorta of *Xenopus* control embryos at stage 33/34, we observed ZO-1 to be expressed between adjacent endothelial cells (Fig. 6A). We additionally observed ZO-1 to be expressed at the cell-cell contact between arterial endothelial cells of EGFL7-depleted embryos indicating that EGFL7 is not required for the formation of tight junctions prior to lumen formation (stage 33/34; Fig. 6B). In control embryos at stage 35/36, we found that ZO-1 becomes distributed to discrete contacts between endothelial cells at the periphery of the aorta lumen thus suggesting that tight junctions are cleared from the cord center to permit lumen formation (Fig. 6C). In contrast, we found that tight junctions persist along the cell-cell contact in the absence of EGFL7 (Fig. 6D) suggesting that EGFL7 acts to clear tight junctions between endothelial cells of the dorsal aorta to allow lumen formation to progress.

Similar to our findings in the aorta, we also observed that ZO-1 is expressed in a punctate pattern between endothelial cells of the posterior cardinal veins at the cell-cell contact of control embryos prior to lumen formation (stage 33/34; Fig. 6E). Likewise, ZO-1 tight junctions form along the cord center between venous endothelial cells of EGFL7-depleted embryos at stage 33/34 (Fig. 6F). In control veins, the junctions are reorganized away from the cord center but maintained at discrete points of cell-cell contact surrounding the lumen by stage 35/36 (Fig. 6G). In contrast, the venous endothelial cells of EGFL7-depleted embryos remain as a cluster and tight junctions are retained along the contact region where the lumen should have formed by stage 35/36 (Fig. 6H). Collectively, these data suggest that EGFL7 is required for clearing tight junctions away from the cord center to the periphery of lumenized arteries and veins.

### Hierarchical tight junction assembly impedes lumen formation in the absence of EGFL7

**Fig 7:**
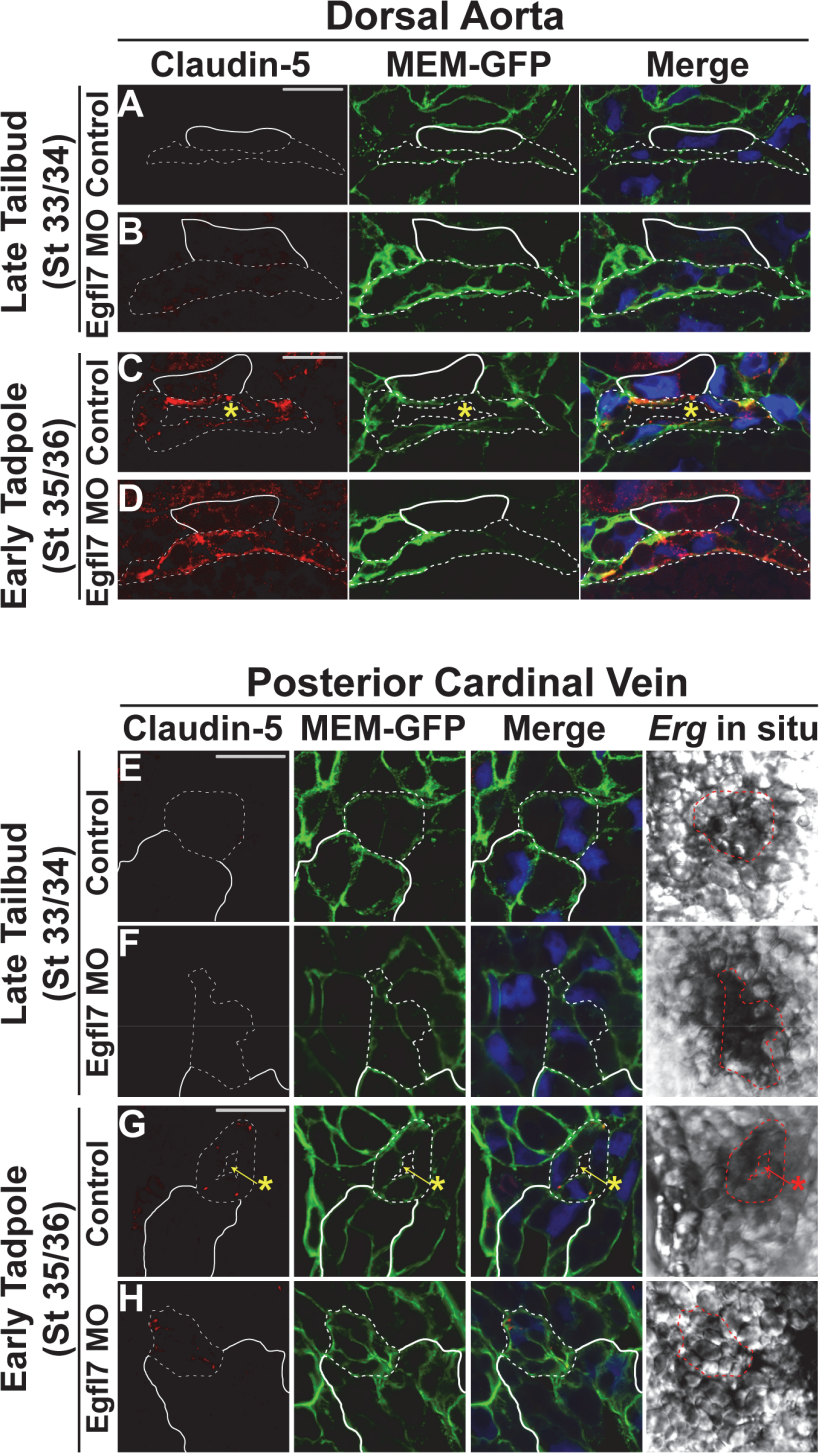
EGFL7 is required for junctional reorganization but not for the hierarchical assembly of tight junctions. (**A-H**). Representative confocal images of transverse sections of the dorsal aorta (DA) and posterior cardinal veins (PCV) stained with Claudin-5 (red), GFP to mark cell membranes (MEM-GFP; green), and DAPI to mark nuclei (blue). Phase contrast images for the PCVs represent the *Erg* in situ signal. All images taken at 63x magnification with scale bars indicating 20 μm. Dotted white and red lines in all panels delineate the endothelial cells comprising each vessel. As anatomical references, the hypochord in (A-D) and the kidney in (E-H) are indicated by solid white lines. (**A-B**). Claudin-5 expression is absent from endothelial cells of the DA in late tailbud stage (stage 33/34) control and EGFL7-depleted embryos. (**C**). Claudin-5 localizes to tight junctions between endothelial cells once the DA lumen has formed in early tadpole stage (stage 35/36) control embryos. (**D**). Claudin-5 expression is apparent in tight junctions between EGFL7-depleted endothelial cells of the DA despite failed lumen formation at early tadpole stage (stage 35/36). (**E-F**). Claudin-5 expression is absent from endothelial cells of the PCV in late tailbud stage (stage 33/34) control and EGFL7-depleted embryos. (**G**). Claudin-5 localizes to distinct tight junctions between endothelial cells around the periphery of the PCV lumen in early tadpole stage (stage 35/36) control embryos. (**H**). Claudin-5 localizes to tight junctions along endothelial cell contacts within the PCV of early tadpole stage (stage 35/36) EGFL7-depleted embryos. 3–4 embryos from each condition/stage were assessed from at least three independent injection batches at the same position along the anterior-posterior axis of the embryo.

To verify our findings with ZO-1, we examined the expression of Claudin-5, a second marker of tight junctions restricted to cell types in the cardiovascular lineage [56,71]. Examination of Claudin-5 expression in the dorsal aorta and posterior cardinal veins reveals that in contrast to ZO-1, Claudin-5 is absent from endothelial tight junctions prior to lumen formation at stage 33/34 in both the aorta and veins of control and EGFL7-depleted embryos (Fig. 7A,B,E,F). However, once lumens have formed in control embryos by stage 35/36, Claudin-5 is distinctly detected at points of cell-cell contact in both the aorta and veins (Fig. 7C,G). These results would therefore imply that endothelial tight junctions are hierarchically assembled, with expression of Claudin-5 following that of ZO-1 to denote more mature vessels. Interestingly, despite the absence of an apparent lumen within the aorta or veins of EGFL7-depleted embryos, Claudin-5 expression can be detected at junctions between adjacent endothelial cells at stage 35/36 (Fig. 7D,H). While the onset of Claudin-5 does not appear to be altered in the absence of EGFL7, these results raise the possibility that EGFL7-depleted endothelial cells may be delayed in either undergoing appropriate cell shape changes or reorganizing cell junctions away from the cord center thus inhibiting the progression of lumen formation.

## Discussion

The transition from cords of endothelial cells into functional vascular tubes is a dynamic morphogenetic process requiring cells to alter their behaviors to promote the expansion of a luminal compartment. Here we have demonstrated some of the cellular and molecular determinants underlying lumen formation of the major vessels in *Xenopus*. Angioblasts that will give rise to the paired posterior cardinal veins and the dorsal aorta are initially specified from the dorsal lateral plate mesoderm. A subset of these angioblasts will go on to migrate towards the midline to form the dorsal aorta while the remaining population will differentiate into the veins. Both arterial and venous endothelial cells are initially found adhered to one another through the formation of ZO-1-containing tight junctions. In addition, the cells exhibit a polygonal, cobblestone-like appearance at this stage further indicative of immature vessels. These initial events appear to be highly conserved as similar cord-like structures have been observed in zebrafish and mouse major vessels [22,56]. In all three species, the formation of an extracellular lumen is accompanied by elongation of cell shape and clearance of junctions away from the cord center. Furthermore in *Xenopus*, we have shown that lumen-containing vessels are also characterized by the expression of Claudin-5 at tight junctions. In zebrafish, ZO-1 expression also precedes Claudin-5 expression therefore suggesting that Claudin-5 marks more mature vessels [56]. However, while Claudin-5 expression was restricted to the arterial lineage in zebrafish [56], we noted its expression in both arteries and veins in *Xenopus*.

### Apicobasal polarity in *Xenopus* vessels

Our findings do however indicate a fundamental difference between early lumen formation in *Xenopus* and other vertebrates. Our studies suggest that apicobasal polarity may not be established during early *Xenopus* vessel formation. Enrichment of makers such as moesin, sialomucins, F-actin, and non-muscle myosin II to the apical surface of endothelial cells is evident as early as the vascular cord stage in mouse and zebrafish [21,22]. However, we fail to ever detect apical expression of aPKCζ or F-actin in vessels prior to or even following lumen formation [63,64,65]. The establishment of apical polarity in mouse and zebrafish vessels has been previously demonstrated to strongly dictate cell behaviors necessary for lumen morphogenesis and proper vessel structure [21,22,30,54]. While further studies with additional apical markers, especially PODXL and moesin, are needed to more clearly deduce the presence or absence of apical polarity in *Xenopus* vessels, the notion of polarity-independent mechanisms underlying lumen formation is not unfounded in biology as a recent zebrafish study demonstrated that endothelial cells collectively migrate to wrap around a luminal compartment and also noted the absence of polarity markers during this novel lumen ensheathment mechanism [25]. Furthermore, in contrast to studies in mouse and zebrafish [26,27,56,59], polarization of fibronectin and laminin to the basal surface is also absent from early *Xenopus* vessels. Collectively, these findings suggest that not only may the establishment of apicobasal polarity be a non-requisite for *Xenous* lumen formation but that the mere emergence of lumens does not constitute a mature vessel with characteristic basal character until a certain amount of remodeling takes place as by late tadpole stages.

If *Xenopus* vessels do arise in a polarity-independent manner, then what is the driving force underlying cell-cell separation in this vertebrate? It remains to be established whether similar mechanisms as cell-cell repulsion in mouse via the CD34 sialomucins exist in *Xenopus* [30,67]. It is also noteworthy that the major vessels in *Xenopus* arise in close association with basement membranes of other organs; for example, the kidney in the case of the posterior cardinal veins and the gut endoderm in the case of the dorsal aorta. It is therefore possible that these adjacent basal surfaces may play some role in the shaping of vascular tubes perhaps by providing a scaffold on which to assemble or in more direct ways by means of cell-cell crosstalk. Our results call for future studies aimed at examining the direct role of polarity, if any, in establishing vascular lumens in *Xenopus*. The continuous generation of specific antibodies in *Xenopus* will also help in addressing the localization of other known apical and basal membrane markers.

### EGFL7 regulates endothelial cell behaviors critical for vascular lumen morphogenesis

We have further delineated the underlying basis for the lumen formation defects observed in EGFL7-depleted embryos. Endothelial cells appeared indistinguishable between control and EGFL7-depleted embryos at late tailbud stage (stage 33/34) before the commencement of lumen formation. Tight junctions formed normally between adjacent endothelial cells and the characteristic polygonal morphology of the cells was indicative of cord-like structures. However, by the time lumens should be generated, EGFL7-depleted endothelial cells fail to elongate and reorganize junctions away from the cord center resulting in the absence of vascular lumens in the majority of cases. These findings are in line with the published literature whereby in EGFL7-depleted zebrafish, angioblasts of the major vessels also fail to reorganize ZO-1-containing junctions and thus remain coalesced together [35]. Although the exact mechanisms underlying endothelial cell shape remain to be elucidated, it has been shown that Rho GTPase signaling is a major factor in modulating the cytoskeleton [72,73]. In fact, in the mouse dorsal aorta, impaired Rho kinase (ROCK) signaling was associated with failure of cells to properly elongate and subsequently form lumens [22]. We have recently demonstrated that EGFL7 lies upstream of the RhoA pathway wherein EGFL7-depleted human endothelial cells exhibit significantly reduced RhoA levels leading to altered cell morphology and aberrant focal adhesion formation [41]. Thus in line with these results, the failure of EGFL7-depleted endothelial cells to undergo proper cell shape changes may be attributed to dysregulation of Rho signaling. However, it still remains to be determined how Rho signaling influences lumen formation in the context of the *Xenopus* embryo.

### Tight junction assembly during lumen formation

The expression of Claudin-5 in tight junctions only upon lumen formation in control embryos implies that it is a marker of maturing vessels. Indeed, studies on Claudin-5 strongly implicate its role in maintaining vascular barrier function, particularly that of the blood-brain-barrier (BBB). In zebrafish, Claudin-5 is highly expressed in endothelial cells of the brain vasculature at a stage when the BBB is established consistent with the presence of a vascular barrier [74,75,76,77]. Furthermore, Claudin-5-null mice do not exhibit any defects in vasculogenesis but at later stages display selective BBB defects further implying that only mature vessels express and require Claudin-5 within tight junctions [78]. Finally, Jin *et al*. and colleagues demonstrated that zebrafish endothelial cells express ZO-1 prior to as well as following tube formation but that the onset of Claudin-5 expression coincides with the emergence of vascular lumens [56]. However, there is also the possibility that the absence of Claudin-5 in early vessels simply implies that tight junctions have not yet formed and that instead ZO-1 localizes to other junctions, namely adherens junctions. There is a precedent for this notion in epithelial cells where ZO-1 has been demonstrated to co-localize with α-catenin in adherens junctions [79,80,81] and even in endothelial cells, where imaging with a ZO-1-expressing transgenic zebrafish line demonstrated some overlap between ZO-1 and the adherens junction marker VE-Cadherin [24]. Indeed it has been suggested that the formation of adherens junctions facilitates tight junction assembly and thus the presence of ZO-1 in early as well as late endothelial junctions may be reflective of its dynamic and variable status [80,82]. It will thus be interesting to examine the nature of ZO-1-expressing junctions during lumen development once more reliable adherens junctions markers (e.g. VE-Cadherin) become available in *Xenopus*.

It is interesting that Claudin-5 becomes expressed in tight junctions of EGFL7-depleted embryos despite the absence of a visible vascular lumen in need of a barrier. Thus, we propose that the onset of Claudin-5 is temporally regulated and its appropriate expression by early tadpole stage (stage 35/36) potentially reflects a delay in EGFL7-depleted endothelial cells from undergoing cell shape changes or reorganizing cell-cell junctions in a timely manner. It may even be plausible that the formation of Claudin-5-expressing tight junctions could further exacerbate the defects associated with EGFL7-depletion as they may in fact further restrict the cells from altering their morphologies or prevent them from redistributing junctions. Consequently, EGFL7-depleted endothelial cells may end up "stuck" together without the ability to generate lumens. ZO-1 expressing transgenic lines have been generated in zebrafish to examine the complex dynamics of junctional reorganization during vessel sprouting and fusion [24,32]. A similar line expressing Claudin-5 would potentially be very useful in looking at the dynamics involved in the transition of a cord of endothelial cells into a functional tube and could provide further insight into the specific role of EGFL7 in mediating the appropriate cellular behaviors necessary for lumen formation.

## Conclusions

We have for the first time demonstrated how vascular lumens arise in the major vessels during *Xenopus* vasculogenesis and have described some of the key steps during this process that depend on EGFL7 function. Vascular lumen formation is a critical process towards the establishment of a functional circulatory system capable of supporting life during embryogenesis. Indeed, the formation of effective and patent vascular tubes is also a current goal of tumor angiogenesis therapy for cancers in which blood flow at the tumor site becomes inefficient due to the abnormal, leaky, tortuous vasculature associated with the tumor microenvironment thus preventing proper delivery of therapeutic agents [83,84]. "Vessel normalization" aims to remodel the tumor vasculature by restoring endothelial cell function and inducing vessel maturation thereby enabling efficient blood flow and minimizing dissemination of cancer cells to sites throughout the body [85,86]. The relationship between EGFL7 and tumor angiogenesis is just beginning to be uncovered and therefore the basic function of this ECM factor, such as that in embryonic lumen formation, may provide new or clearer insights into the mechanisms by which EGFL7 functions during tumorigenesis and how these mechanisms can be harnessed to generate effective therapeutics.

## Supporting Information

S1 Fig EGFL7 is not required for ECM deposition. (**A-D**). Representative confocal images of transverse sections of the dorsal aorta (DA) and posterior cardinal veins (PCV) stained with fibronectin (red), GFP to mark cell membranes (MEM-GFP; green), and DAPI to mark nuclei (blue). All images taken at 63x magnification with scale bars indicating 20 μm. Dotted white and red lines in all panels delineate the endothelial cells comprising each vessel. As anatomical references, the hypochord in (A-B) and the kidney in (C-D) are indicated by solid white lines. Asterisks denote the vessel lumen. (**A**) Fibronectin does not exclusively localize to the basal surface of the dorsal aorta in early tadpole stage (stage 35/36) control embryos but some apical deposits are apparent (arrowheads). (**B**) A similar distribution pattern for fibronectin is observed in the dorsal aorta of early tadpole stage (stage 35/36) EGFL7-depleted embryos. (**C**) Enrichment of fibronectin to the basal surface of the posterior cardinal vein is not apparent in early tadpole stage (stage 35/36) control embryos but there is some expression on the apical surface (arrowheads). (**D**) Failure of lumens to form by early tadpole stage (stage 35/36) EGFL7-depleted embryos is not characterized by aberrant deposition of fibronectin around endothelial cells. 3–4 embryos from each condition/stage were assessed from at least three independent injection batches at the same position along the anterior-posterior axis of the embryo.
(TIF)

S1 Table List of antibodies used in immunohistochemistry. Antibody name is accompanied by catalog number and company purchased from as well as dilution used.
(DOCX)
