# Supplementary figures and images for: A Distinct Mechanism of Vascular Lumen Formation in Xenopus Requires EGFL7
Source: PLoS One. 2015 Feb 23;10(2):e0116086. doi: 10.1371/journal.pone.0116086 (PMC4338030; doi:10.1371/journal.pone.0116086)

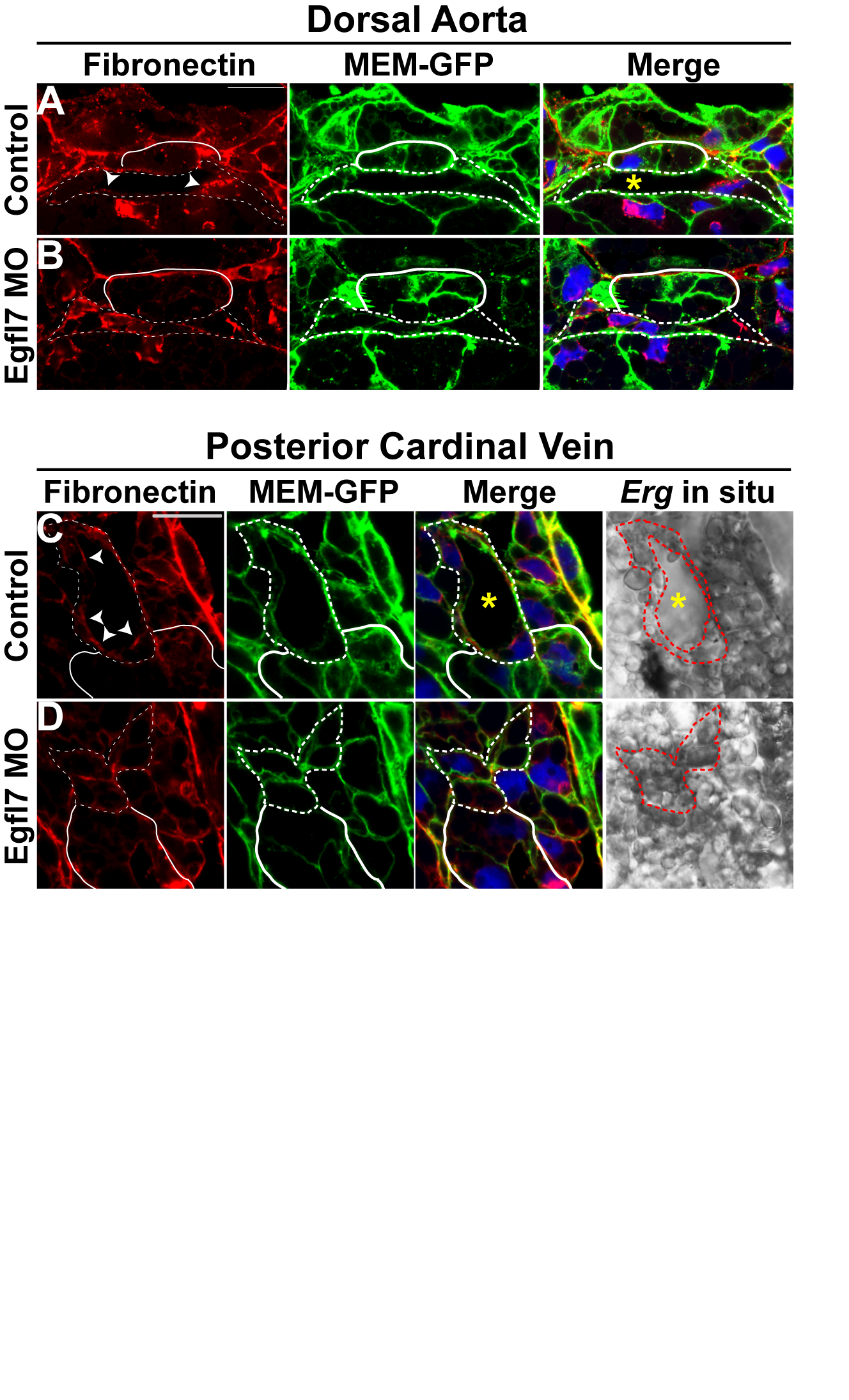

Supplement: S1 Fig — (A-D). Representative confocal images of transverse sections of the dorsal aorta (DA) and posterior cardinal veins (PCV) stained with fibronectin (red), GFP to mark cell membranes (MEM-GFP; green), and DAPI to mark nuclei (blue). All images taken at 63x magnification with scale bars indicating 20 μm. Dotted white and red lines in all panels delineate the endothelial cells comprising each vessel. As anatomical references, the hypochord in (A-B) and the kidney in (C-D) are indicated by solid white lines. Asterisks denote the vessel lumen. (A) Fibronectin does not exclusively localize to the basal surface of the dorsal aorta in early tadpole stage (stage 35/36) control embryos but some apical deposits are apparent (arrowheads). (B) A similar distribution pattern for fibronectin is observed in the dorsal aorta of early tadpole stage (stage 35/36) EGFL7-depleted embryos. (C) Enrichment of fibronectin to the basal surface of the posterior cardinal vein is not apparent in early tadpole stage (stage 35/36) control embryos but there is some expression on the apical surface (arrowheads). (D) Failure of lumens to form by early tadpole stage (stage 35/36) EGFL7-depleted embryos is not characterized by aberrant deposition of fibronectin around endothelial cells. 3–4 embryos from each condition/stage were assessed from at least three independent injection batches at the same position along the anterior-posterior axis of the embryo. (TIF) [file pone.0116086.s001.tif]
