# Supplementary material for: A Distinct Mechanism of Vascular Lumen Formation in Xenopus Requires EGFL7
Source: PLoS One. 2015 Feb 23;10(2):e0116086. doi: 10.1371/journal.pone.0116086 (PMC4338030; doi:10.1371/journal.pone.0116086)
Supplement: S1 Table — Antibody name is accompanied by catalog number and company purchased from as well as dilution used. (DOCX) [file pone.0116086.s002.docx]

| **Antibody** | **Catalog Number** | **Dilution Used** |
| --- | --- | --- |
| GFP | Molecular Probes, A6455 | 1:1000 |
| GFP (JL8) | Clontech, 632381 | 1:1000 |
| ZO-1 | Invitrogen, 33-9100 | 1:100 |
| Claudin-5 | Santa Cruz Biotechnologies, sc-28670 | 1:500 |
| Laminin | Sigma, L9393 | 1:100 |
| Fibronectin | Sigma, F3648 | 1:250 |
| Atypical PKCζ | Santa Cruz Biotechnologies, sc-216 | 1:500 |
| Alexa Fluor 488 Goat anti-Mouse | Molecular Probes, A11001 | 1:1000 |
| Alexa Fluor 488 Donkey anti-Rabbit | Molecular Probes, A21206 | 1:1000 |
| Alexa Fluor 546 Goat anti-Mouse | Molecular Probes, A21123 | 1:1000 |
| Alexa Fluor 546 Goat anti-Rabbit | Molecular Probes, A11010 | 1:1000 |

**Supplemental Table 1.** List of antibodies used in immunohistochemistry.
